# The prognostic values of signal transducers activators of transcription family in ovarian cancer

**DOI:** 10.1042/BSR20170650

**Published:** 2017-07-17

**Authors:** Saisai Li, Bo Sheng, Menghuang Zhao, Qi Shen, Haiyan Zhu, Xueqiong Zhu

**Affiliations:** Department of Obstetrics and Gynecology, The Second Affiliated Hospital of Wenzhou Medical University, Wenzhou 325027, China

**Keywords:** KM plotter, Ovarian cancer, Prognosis, STATs

## Abstract

Signal transducer and activator of transcription (STAT), a family of latent cytoplasmic transcription factors, are composed of seven identified members (STAT1, STAT2, STAT3, STAT4, STAT5a, STAT5b, STAT6). STATs are associated with several biological processes such as cell proliferation, invasion, and metastasis in various cancer types. In addition, the STAT family has been well studied as a prognostic predictor for a considerable number of solid tumors. However, the prognostic value of the STAT family in ovarian cancer patients remains unclear. In our present study, we intend to access the prognostic roles of the STAT family in ovarian carcinoma through the ‘Kaplan–Meier plotter’ (KM plotter) online database, which collected gene expression data and survival information (overall survival (OS)) from a total of 1582 ovarian cancer patients. Our results show that high mRNA expression of *STAT1, STAT4, STAT5a, STAT5b*, and *STAT6*, are correlated to a better OS of ovarian cancer patients, especially the high level of *STAT1* and *STAT4* are significantly related to a favorable OS for serous ovarian cancer patients. We further accessed the prognostic roles of individual *STATs* in other clinicopathological features, such as pathological grades, clinical stages, and TP53 mutation, and found that these genes indicate a favorable prognosis especially for late stage, poor differentiation, and TP53 mutated ovarian cancer patients. In conclusion, these results suggest that the STAT family plays a significant prognostic role in ovarian carcinoma and individual STATs, except STAT2 and STAT3, may act as favorable prognostic markers in ovarian cancer.

## Introduction

Ovarian cancer is the fifth most lethal malignancy amongst women diagnosed with cancers in the United States with approximately 14240 cancer-related deaths from this disease annually [1]. Combination of surgery and chemotherapy is the standard treatment for the advanced stage in ovarian cancer [2]. Despite considerable efforts to improve early detection and advances in chemotherapy, the 5-year survival rate remains only 30% [3]. Identification of favorable prognostic biomarkers for ovarian cancer, in some ways, could improve clinical outcomes of these patients.

Signal transducer and activator of transcription (STAT), a family of latent cytoplasmic transcription factors, is composed of seven identified members (STAT1, STAT2, STAT3, STAT4, STAT5a, STAT5b, STAT6) [4]. These genes are activated in response to cytokines, hormones, and growth factors [4-6]. Ligand-dependent activation of STATs, which is a transient process lasting from minutes to hours, plays important roles in regulating cell proliferation, cellular transformation, tumor formation, and immune responses and is also involved in tumorigenesis, metastasis, and angiogenesis [4-7]. Indeed, previous studies have shown that STATs were constitutively activated in ovarian cancer and played a pivotal role in oncogenesis of this disease [8,9].

Since STAT family plays an important role in the initiation and development of cancers, these genes have been well studied as a prognostic predictor for a considerable number of solid tumors [10-22]. However, the prognostic value of the STAT family in ovarian cancer patients is limited and the results remain controversial [23-26]. In the present study, we first comprehensively explored the prognostic significance of seven *STAT* genes in patients with ovarian carcinoma by using the Kaplan–Meier plotter (KM plotter).

## Materials and methods

### KM plotter database

An online database (http://kmplot.com/analysis/) [27] was used to investigate the association between individual* STAT* mRNA levels and overall survival (OS) of ovarian cancer patients. Currently, this database is capable of assessing the effect of 54675 genes of survival in breast cancer [27], ovarian cancer [28], lung cancer [29], as well as gastric cancer data. In this database, gene expression data and OS information of 1582 ovarian cancer patients were downloaded from Gene Expression Omnibus, Cancer Biomedical Informatics Grid, and The Cancer Genome Atlas cancer datasets [29]. Additionally, they offered clinical data, such as histology, grade, stage, TP53 mutation status, and treatment of ovarian cancer patients.

In simpler terms, seven *STAT* members (*STAT1, STAT2, STAT3, STAT4, STAT5a, STAT5b*, and *STAT6*) were entered into the database (http://kmplot.com/analysis/index.php?p=service&cancer=ovar) to get Kaplan–Meier survival plots. The expression cut-off points of individual *STAT* genes were determind according to their median mRNA levels amongst the selected ovarian cancer samples. *STAT* expression status were finally classified into ‘low’ and ‘high’ according to the comparisons between expression values with established cutoffs. The two patient cohorts were compared with a Kaplan–Meier survival plot, and then hazard ratio (HR), 95% confidence intervals (CIs), and log-rank *P* were determined and presented on internet.

## Results

**Figure 1:**
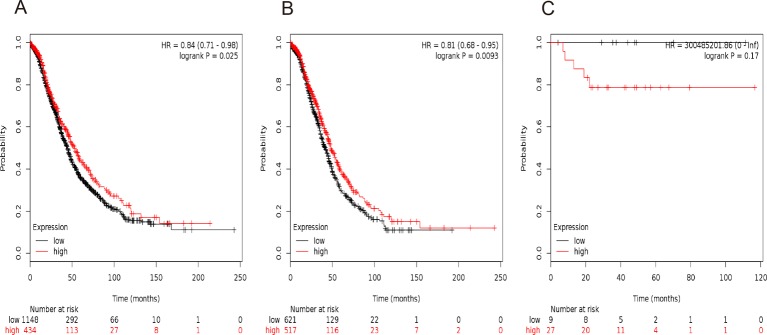
The prognostic value of *STAT1* expression in ovarian cancer. The prognostic value of *STAT1* expression in www.kmplot.com. Affymetrix ID for *STAT1*: 200887_s_at. OS curves are plotted for (**A**) all the patients (*n*=1582), (**B**) serous cancer patients (*n*=1138), and (**C**) endometrioid cancer patients (*n*=36).

In our present study, all the seven *STAT* members’ Kaplan–Meier survival information can be determined on www.kmplot.com. We initially evaluated the prognostic value of *STAT1* in the database. Affymetrix IDs for *STAT1*: 200887_s_at. OS curves were plotted for all the ovarian cancer patients (*n*=1582) (Figure 1A), for serous cancer patients (*n*=1138) (Figure 1B), and for endometrioid cancer patients (*n*=36) (Figure 1C). High mRNA expression of *STAT1* was related to a better OS in all the ovarian cancer patients, HR =0.84 (0.71−0.98), *P*=0.025, especially for serous ovarian cancer patients, HR =0.81 (0.68−0.95), *P*=0.0093, but not in endometrioid cancer patients, HR =300485201.86 (0−Inf), *P*=0.17.

**Figure 2:**
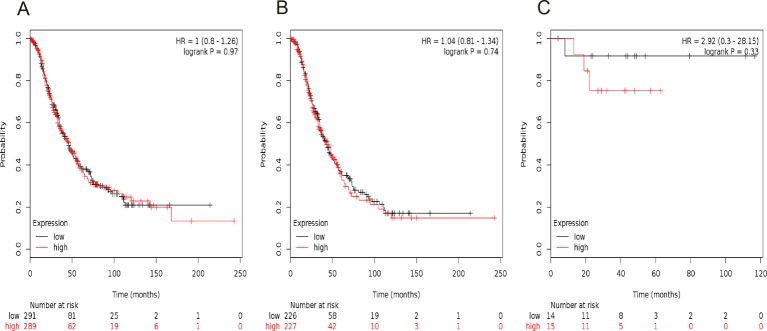
The prognostic value of *STAT2* expression in ovarian cancer. The prognostic value of *STAT2* expression in www.kmplot.com. Affymetrix ID for *STAT2*: 225636_at. OS curves are plotted for (**A**) all the patients (*n*=580), (**B**) serous cancer patients (*n*=453), and (**C**) endometrioid cancer patients (*n*=29).

We then evaluated the prognostic significance of *STAT2* mRNA expression in the database. Affymetrix IDs for *STAT2*: 225636_at. *STAT2* mRNA level showed a null association with OS amongst all the ovarian cancer patients, HR =1.00 (0.80−1.26), *P*=0.97 (Figure 2A), serous ovarian cancer patients, HR =1.04 (0.81−1.34), *P*=0.74 (Figure 2B), as well as endometrioid ovarian cancer patients, HR =2.92 (0.30−28.15), *P*=0.33 (Figure 2C).

**Figure 3:**
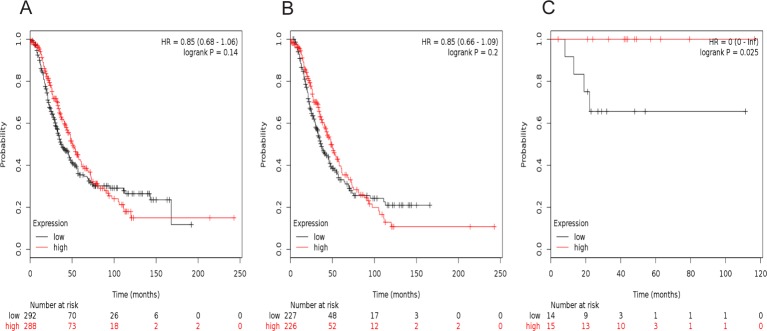
The prognostic value of *STAT3* expression in ovarian cancer. The prognostic value of *STAT3* expression in www.kmplot.com. Affymetrix ID for *STAT3*: 225289_at. OS curves are plotted for (**A**) all the patients (*n*=580), (**B**) serous cancer patients (*n*=453), and (**C**) endometrioid cancer patients (*n*=29).

Figure 3 showed the prognostic value of *STAT3* in the database. Affymetrix IDs for *STAT3*: 225289_at. Increased *STAT3* mRNA expression had no effect on OS for all ovarian cancer patients, HR =0.85 (0.68–1.06), *P*=0.14 (Figure 3A), as well as serous cancer patients, HR =0.85 (0.66–1.09), *P*=0.2 (Figure 3B). However, *STAT3* mRNA expression predicted a better OS in 29 endometrioid cancer patients, HR =0 (0−Inf), *P*=0.025 (Figure 3C).

**Figure 4:**
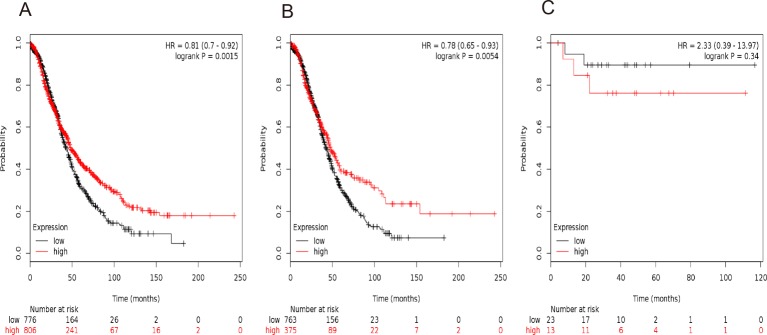
The prognostic value of *STAT4* expression in ovarian cancer. The prognostic value of *STAT4* expression in www.kmplot.com. Affymetrix ID for *STAT4*: 206118_at. OS curves are plotted for (**A**) all the patients (*n*=1582), (**B**) serous cancer patients (*n*=1138), and (**C**) endometrioid cancer patients (*n*=36).

Figure 4 showed the prognostic value of *STAT4* in the database. Affymetrix IDs for *STAT4*: 206118_at. Overexpression of *STAT4* mRNA was significantly related to a favorable OS for all the ovarian cancer patients, HR =0.81 (0.70−0.92), *P*=0.0015 (Figure 4A), serous cancer patients, HR =0.78 (0.65−0.93), *P*=0.0054 (Figure 4B). However, *STAT4* mRNA expression was uncorrelated with OS in endometrioid cancer patients, HR =2.33 (0.39−13.97), *P*=0.34 (Figure 4C).

**Figure 5:**
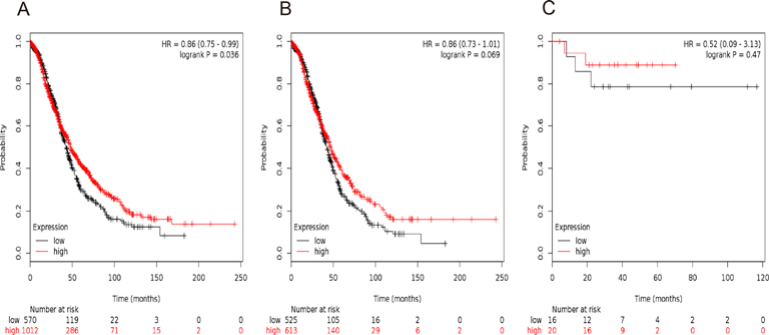
The prognostic value of *STAT5a* expression in ovarian cancer. The prognostic value of *STAT5a* expression in www.kmplot.com. Affymetrix ID for *STAT5a*: 203010_at. OS curves are plotted for (**A**) all the patients (*n*=1582), (**B**) serous cancer patients (*n*=1138), and (**C**) endometrioid cancer patients (*n*=36).

**Figure 6:**
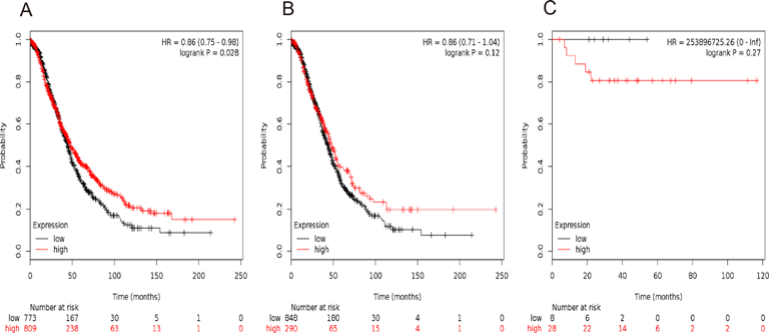
The prognostic value of *STAT5b* expression in ovarian cancer. The prognostic value of *STAT5b* expression in www.kmplot.com. Affymetrix ID for *STAT5b*: 212549_at. OS curves are plotted for (**A**) all the patients (*n*=1582), (**B**) serous cancer patients (*n*=1138), and (**C**) endometrioid cancer patients (*n*=36).

Figures 5 and 6 presented the prognostic significance of *STAT5a* and *STAT5b*, respectively. Affymetrix IDs were as following: 203010_at (*STAT5a*) and 212549_at (*STAT5b*). Both elevated *STAT5a* and *STAT5b* mRNA expression are associated with a favorable OS for all the ovarian cancer patients (*STAT5a*: *P*=0.036, Figure 5A; *STAT5b*: *P*=0.028, Figure 6A). Nevertheless, with regard to serous ovarian cancer patients and endometrioid ovarian cancer patients, there was no significant difference in HR estimates between study strata (Figure 5 B,5C and Figure 6B,6C).

**Figure 7:**
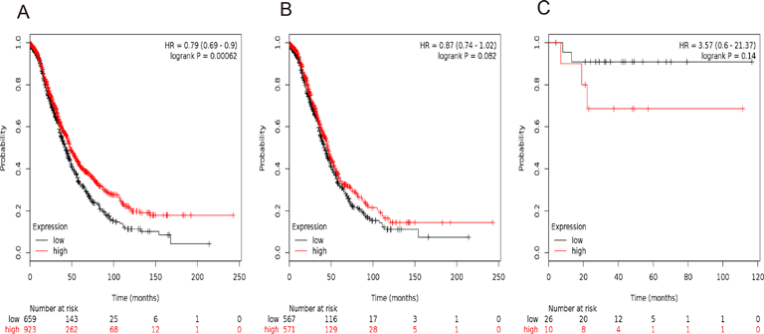
The prognostic value of *STAT6* expression in ovarian cancer. The prognostic value of *STAT6* expression in www.kmplot.com. Affymetrix ID for *STAT6*: 201331_s_at. OS curves are plotted for (**A**) all the patients (*n*=1582), (**B**) serous cancer patients (*n*=1138), and (**C**) endometrioid cancer patients (*n*=36).

Finally, we investigated the prognostic significance of *STAT6* mRNA expression in the database. Affymetrix IDs for *STAT6*: 201331_s_at. High levels of *STAT6* mRNA were significantly correlated to a favorable OS for all the ovarian cancer patients, HR =0.79 (0.69−0.9), *P*=0.00062 (Figure 7A). Nevertheless, *STAT6* showed no effect on OS either amongst serous cancer patients, HR =0.87 (0.74−1.02), *P*=0.082 (Figure 7B) or endometrioid cancer patients, HR =3.57 (0.6−21.37), *P*=0.14(Figure 7C).

To further explore the correlation of individual *STAT* with other clinicopathological features, we investigated the correlation with pathological grades (Table 1), clinical stages (Table 2), TP53 mutation (Table 3) of ovarian cancer patients. As shown in Table 1, high expression of *STAT1, STAT3, STAT4*, and *STAT6* mRNA were correlated to a better OS in grade III ovarian cancer patients, but not in grade I or II ovarian cancer patients (Table 1).

**Table 1:** Correlation of *STAT* gene expression level with OS in different pathological grades in ovarian cancer patients

STAT	Pathological grade	Cases	HR (95% CI)	*P*-value
STAT1	I	56	2.19 (0.84−5.76)	0.1
	II	315	1.32 (0.95−1.83)	0.1
	III	968	0.73 (0.61−0.87)	0.00039*
STAT2	I	41	1.91 (0.59−6.16)	0.27
	II	153	1.33 (0.83−2.14)	0.24
	III	344	1 (0.76−1.31)	0.98
STAT3	I	41	0.67 (0.22–2.02)	0.47
	II	153	1.03 (0.65–1.63)	0.89
	III	344	0.75 (0.57–0.99)	0.039*
STAT4	I	56	2.23 (0.85−5.89)	0.096
	II	315	0.76 (0.53−1.07)	0.12
	III	968	0.73 (0.62−0.87)	0.00044*
STAT5a	I	56	2.05 (0.67−6.25)	0.2
	II	315	0.79 (0.58−1.08)	0.14
	III	968	0.88 (0.74−1.04)	0.13
STAT5b	I	56	1.66 (0.59−4.7)	0.34
	II	315	0.81 (0.59−1.1)	0.17
	III	968	0.84 (0.69−1.03)	0.088
STAT6	I	56	6.39 (1.82−22.44)	0.00094*
	II	315	0.8 (0.57−1.13)	0.2
	III	968	0.74 (0.6−0.9)	0.003*

*P<0.05.

**Table 2:** Correlation of *STAT* gene expression level with OS in different clinical stage ovarian cancer patients

STAT	Clinical stages	Cases	HR (95% CI)	*P*-value
STAT1	I + II	133	2.53 (0.76−8.46)	0.12
	III + IV	1148	0.82 (0.7−0.96)	0.015*
STAT2	I + II	81	0.65 (0.22−1.95)	0.44
	III + IV	414	0.97 (0.75−1.25)	0.8
STAT3	I + II	81	1.1 (0.39–3.17)	0.85
	III + IV	414	0.89 (0.69−1.15)	0.37
STAT4	I + II	133	0.49 (0.22−1.1)	0.078
	III + IV	1148	0.71 (0.6−0.83)	0*
STAT5a	I + II	133	0.33 (0.15−0.73)	0.0038*
	III + IV	1148	0.75 (0.63−0.9)	0.0014*
STAT5b	I + II	133	0.42 (0.17−1)	0.044*
	III + IV	1148	0.78 (0.67−0.92)	0.0024*
STAT6	I + II	133	0.36 (0.16−0.8)	0.0087*
	III + IV	1148	0.76 (0.63−0.91)	0.0028*

*P<0.05.

**Table 3:** Correlation of *STAT* genes expression level with OS in ovarian cancer patients with different TP53 mutation status

STAT	TP53 mutation	Cases	HR (95% CI)	*P*-value
STAT1	No	86	0.67 (0.37−1.24)	0.2
	Yes	439	0.73 (0.56−0.94)	0.013*
STAT2	No	11	Not available	Not available
	Yes	57	0.84 (0.48−1.47)	0.53
STAT3	No	11	Not available	Not available
	Yes	57	0.41 (0.23−0.73)	0.0018*
STAT4	No	86	0.61 (0.33−1.12)	0.11
	Yes	439	0.87 (0.68−1.12)	0.28
STAT5a	No	86	1.82 (0.96−3.46)	0.063
	Yes	439	1.21 (0.92−1.59)	0.16
STAT5b	No	86	1.75 (0.94−3.24)	0.073
	Yes	439	1.27 (0.98−1.65)	0.065
STAT6	No	86	1.56 (0.8−3.02)	0.19
	Yes	439	1.45 (1.07−1.97)	0.016*

*P<0.05.

With regard to clinical stages, while *STAT5a, STAT5b*, and *STAT6* were related to a positive OS both in stages I/II and III/IV ovarian cancer patients, *STAT1* and *STAT4* were correlated to a favorable OS in stages III and IV ovarian cancer patients (Table 2).

We further investigated association between individual *STATs* and prognosis according to the TP53 status, *STAT1, STAT3*, and *STAT6* indicated an improved OS in TP53 mutated ovarian cancer patients (Table 3).

## Discussion

In our present study, we comprehensively investigated the prognostic value of seven *STAT* members in ovarian cancer patients by using the KM plotter database. Amongst the seven genes, only *STAT2* and *STAT3* showed no effect on the OS of ovarian cancers, i.e. high mRNA expression of *STAT1, STAT4, STAT5a, STAT5b*, and *STAT6* was correlated to a better OS for ovarian cancer patients, especially the high level of *STAT1* and *STAT4* was significantly related to a favorable OS for serous ovarian cancer patients.

A number of studies have reported the relationship between STAT1 expression and the prognosis in various types of cancers; however, the results are controversial. Increased STAT1 expression and high STAT1 activation (p-STAT1 protein levels) were related to a favorable prognosis in colorectal carcinoma [10,11], oral squamous cell carcinoma [12], as well as in breast cancer [13]. In addition, Sun et al. [14] observed that overexpression of STAT1 was inversely related to malignant behaviors (lymph node metastasis, tumor dedifferentiation, advanced stage) of pancreatic cancer. And they believed that the silencing of STAT1 expression correlated with poor patient survival and performance [14]. But so far, studies about STAT1 in ovarian cancer are limited. Our results showed that high expression of *STAT1* mRNA was correlated to a better OS for all the ovarian cancer patients, particularly for serous cancer patients. This result is in-line with previous result. In high-grade serous ovarian cancer (HGSC), higher STAT1 expression correlated with increased progression-free survival and predicted better prognosis [23]. In addition, we observed that high mRNA expression of *STAT1* indicated a better OS in grade III and stage III/IV ovarian cancer patients, but not in grade I/II and stage I/II ovarian cancer patients, suggesting that STAT1 is a favorable prognostic marker for ovarian cancer patients, especially for late stage and poor differentiation serous ovarian patients.

STAT2 is unique in the STAT family because it was activated only by interferon [30]. However, little is known about the function of STAT2 in malignancies. In the present study, we found that *STAT2* mRNA levels had no effect on the prognosis of ovarian cancer patients.

STAT3, which can be triggered by cytokines and growth factors [31,32], was constitutively activated in some types of cancer [10,15,16]. It was not only involved in cancer development and progression but also contributed to their survival. STAT3 was overexpressed in colorectal carcinoma and their overexpression contributed to an increase in median OS by approximately 30 months [10]. Grabner et al. [33] detected STAT3 may act as a tumor suppressor through STAT3-NF-κB-IL-8 axis in KRAS mutant lung adenocarcinoma and a molecular marker with favorable prognostic value. In the same line of evidence, it was shown that p-STAT3 was reported as a favorable prognosis marker in breast cancer [15], head and neck squamous cell carcinoma [16], and leiomyosarcoma [34]. Amongst the *STAT* family, *STAT3* is the most studied *STAT* in ovarian cancer. Previous studies have demonstrated that *p-STAT3* was associated with poor survival in ovarian cancer [24,25]. Rosen et al. [24] detected that the activation and translocation of *p-STAT3* to the nucleus were frequent events in ovarian carcinoma, which were associated with a poor prognosis. Yang et al. [25] reported that increased *p-STAT3* expression in omental tissue was associated with poor survival amongst patients with high-grade epithelial ovarian cancer. Intriguingly, our study shows a null association between *STAT3* mRNA expression and OS of ovarian cancer patients. This can be attributed to different study design, detection method (while previous studies measured protein levels, our study was involved in mRNA levels) [24,25], specimens (Yang et al. [25] using omental tissue), sample size, and cutoff. Therefore, we suggest that the mRNA level of *STAT3* in ovarian tissues shows no effect on OS of ovarian cancers.

STAT4 is one of the STAT family members that specifically mediates IL-12 signaling, affecting a wide range of immune cell physiology [35]. Similar to STAT2, the study about STAT4 in malignancies is limited. Wang et al. [17] demonstrated that STAT4 expression is an independent marker of favorable prognosis and may act as a tumor suppressor in hepatocellularcarcinoma (HCC). To the best of our knowledge, this is the first report investigating the relationship between STAT4 and prognosis of ovarian cancer. In this report, our results show that higher mRNA levels of *STAT4* are significantly correlated to a better OS for all the ovarian cancer patients, especially serous cancer patients. Furthermore, we found that *STAT4* mRNA levels predicted an improved OS in grade III and stage III/IV but not grade I/II and stage I/II ovarian cancer patients, suggesting that this gene is a favorable prognosis indicator especially for late stage and poor differentiation in ovarian cancer patients.

Compared with other STATs, STAT5 consists of two highly homologous isoforms, STAT5a and STAT5b [4]. Although the prognostic role of *STAT5* genes (*STAT5a* and *STAT5b*) varied in different types of cancers, the majority of studies reported that *STAT5* genes predicted a favorable clinical outcome in malignancies. Chen et al. [18] reported that constitutive STAT5 activation was associated with a better survival in cervical carcinoma patients who have accepted radiation therapy. Overexpression of STAT5 in lung cancer was reported to be a positive prognostic marker for patients treated with surgery [19]. Similar results were observed in human breast cancer, i.e. overexpression and overactivation of STAT5 indicated a favorable prognosis of breast cancer [20-22]. Our current results show that high mRNA expression of *STAT5a* and *STAT5b* related to an improved OS of ovarian cancer, which is consistent with only previous study to explore the impact of STAT5 on the prognosis of ovarian cancer patients [26].

STAT6 has a dual role as signaling molecule and transcription factor [36]. It is tightly connected to IL-4 and IL-13 signaling, and plays a key role in TH2 polarization of the immune system [36]. The study about the prognostic value of STAT6 expression in malignancies is limited. So far, the association between STAT6 and cancer patients’ clinical outcomes was only investigated in colorectal cancer. Wang et al. [37] examined 119 colorectal cancer patients by immunohistochemistry and found patients with STAT6-positive expression had lower survival rates than those with STAT6-negative expression. In the present study, we found that high mRNA expression of *STAT6* was correlated to a better OS for all the ovarian cancer patients, suggesting STAT6 has different prognostic significance across cancer types. Furthermore, high mRNA expression of STAT6 was correlated to a better OS in grade III ovarian cancer patients, but not in grade I or II ovarian cancer patients, implying that STAT6 may be a favorable prognostic indictor especially for poor differentiation ovarian cancer.

Interestingly, when we further explored the prognostic roles of individual *STATs* in TP53 mutation, our data shows that *STAT1, STAT3*, and *STAT6*’s high mRNA expression indicated a better OS in TP53 mutated, but not in TP53 wild-type ovarian cancer patients. These observations indicate that STATs family may be favorable prognosis indicators for TP53 mutated ovarian cancer patients. Considering the small sample size of TP53 wild-type ovarian cancer patients, we can not determine the prognostic value of STATs family in these subtype ovarian cancer, further study with large sample size are needed.

## Conclusion

In summary, our results show that high mRNA expression of all the individual *STATs* except *STAT2* and *STAT3* are correlated to a better OS for all the ovarian cancer patients, especially the high level of *STAT1* and *STAT4* are significantly related to a favorable OS for serous ovarian cancer patients. And we also found that these genes indicated a favorable prognosis especially for late stage, poor differentiation, and TP53 mutated ovarian cancer patients. These results indicate that STATs family plays a significantly prognostic role in ovarian carcinoma and individual STATs, except STAT2 and STAT3, and may act as a favorable prognostic marker in ovarian cancer. Although our data were statistically significant, the relationship between individual STATs expression and the prognosis of ovarian cancer needs further exploration. Our further studies will validate these results at the in situ protein epression level in human ovarian cancer samples and explore the clinical application of STAT family in ovarian cancer treatment.

## Abbreviations

CI: confidence interval
HR: hazard ratio
IL: interleukin
KM plotter: Kaplan–Meier plotter
KRAS: kirsten rat saccoma viral oncogene
OS: overall survival
STAT: signal transducer and activator of transcription
TP53: tumor protein p53
TH2: T helper cells 2

## References

[1] Siegel R.L., Miller K.D. and Jemal A. (2016) Cancer statistics, 2016. CA Cancer J. Clin. 66, 7–30 26742998 10.3322/caac.21332

[2] Vergote I., Trope C.G., Amant F., Kristensen G.B., Ehlen T., Johnson N. (2010) Neoadjuvant chemotherapy or primary surgery in stage IIIC or IV ovarian cancer. N. Engl. J. Med. 363, 943–953 20818904 10.1056/NEJMoa0908806

[3] Coleman M.P., Forman D., Bryant H., Butler J., Rachet B., Maringe C. (2011) Cancer survival in Australia, Canada, Denmark, Norway, Sweden, and the UK, 1995-2007 (the International Cancer Benchmarking Partnership): an analysis of population-based cancer registry data. Lancet 377, 127–138 21183212 10.1016/S0140-6736(10)62231-3 PMC3018568

[4] Darnell J.E. Jr (1997) STATs and gene regulation. Science 277, 1630–1635 9287210 10.1126/science.277.5332.1630

[5] Darnell J.E. Jr, Kerr I.M. and Stark G.R. (1994) Jak-STAT pathways and transcriptional activation in response to IFNs and other extracellular signaling proteins. Science 264, 1415–1421 8197455 10.1126/science.8197455

[6] Levy D.E. and Darnell J.E. Jr (2002) Stats: transcriptional control and biological impact. Nat. Rev. Mol. Cell Biol. 3, 651–662 12209125 10.1038/nrm909

[7] Mui A.L. (1999) The role of STATs in proliferation, differentiation, and apoptosis. Cell. Mol. Life Sci. 55, 1547–1558 10526572 10.1007/s000180050394 PMC11146798

[8] Landen C.N. Jr, Lin Y.G., Armaiz Pena G.N., Das P.D., Arevalo J.M., Kamat A.A. (2007) Neuroendocrine modulation of signal transducer and activator of transcription-3 in ovarian cancer. Cancer Res. 67, 10389–10396 17974982 10.1158/0008-5472.CAN-07-0858

[9] Huang M., Page C., Reynolds R.K. and Lin J. (2000) Constitutive activation of stat 3 oncogene product in human ovarian carcinoma cells. Gynecol. Oncol. 79, 67–73 11006034 10.1006/gyno.2000.5931

[10] Gordziel C., Bratsch J., Moriggl R., Knosel T. and Friedrich K. (2013) Both STAT1 and STAT3 are favourable prognostic determinants in colorectal carcinoma. Br. J. Cancer 109, 138–146 23756862 10.1038/bjc.2013.274 PMC3708576

[11] Simpson J.A., Al-Attar A., Watson N.F., Scholefield J.H., Ilyas M. and Durrant L.G. (2010) Intratumoral T cell infiltration, MHC class I and STAT1 as biomarkers of good prognosis in colorectal cancer. Gut 59, 926–933 20581241 10.1136/gut.2009.194472

[12] Laimer K., Spizzo G., Obrist P., Gastl G., Brunhuber T., Schafer G. (2007) STAT1 activation in squamous cell cancer of the oral cavity: a potential predictive marker of response to adjuvant chemotherapy. Cancer 110, 326–333 17559122 10.1002/cncr.22813

[13] Widschwendter A., Tonko-Geymayer S., Welte T., Daxenbichler G., Marth C. and Doppler W. (2002) Prognostic significance of signal transducer and activator of transcription 1 activation in breast cancer. Clin. Cancer Res. 8, 3065–3074 12374673

[14] Sun Y., Yang S., Sun N. and Chen J. (2014) Differential expression of STAT1 and p21 proteins predicts pancreatic cancer progression and prognosis. Pancreas 43, 619–623 24658320 10.1097/MPA.0000000000000074

[15] Aleskandarany M.A., Agarwal D., Negm O.H., Ball G., Elmouna A., Ashankyty I. (2016) The prognostic significance of STAT3 in invasive breast cancer: analysis of protein and mRNA expressions in large cohorts. Breast Cancer Res. Treat. 156, 9–20 26907764 10.1007/s10549-016-3709-z

[16] Pectasides E., Egloff A.M., Sasaki C., Kountourakis P., Burtness B., Fountzilas G. (2010) Nuclear localization of signal transducer and activator of transcription 3 in head and neck squamous cell carcinoma is associated with a better prognosis. Clin. Cancer Res. 16, 2427–2434 20371693 10.1158/1078-0432.CCR-09-2658 PMC3030188

[17] Wang G., Chen J.H., Qiang Y., Wang D.Z. and Chen Z. (2015) Decreased STAT4 indicates poor prognosis and enhanced cell proliferation in hepatocellular carcinoma. World J. Gastroenterol. 21, 3983–3993 25852285 10.3748/wjg.v21.i13.3983 PMC4385547

[18] Chen H.H., Chou C.Y., Wu Y.H., Hsueh W.T., Hsu C.H., Guo H.R. (2012) Constitutive STAT5 activation correlates with better survival in cervical cancer patients treated with radiation therapy. Int. J. Radiat. Oncol. Biol. Phys. 82, 658–666 21300446 10.1016/j.ijrobp.2010.11.043

[19] He Y., Zhou Z., Hofstetter W.L., Zhou Y., Hu W., Guo C. (2012) Aberrant expression of proteins involved in signal transduction and DNA repair pathways in lung cancer and their association with clinical parameters. PLoS ONE 7, e31087 22348039 10.1371/journal.pone.0031087 PMC3277494

[20] Sultan A.S., Xie J., LeBaron M.J., Ealley E.L., Nevalainen M.T. and Rui H. (2005) Stat5 promotes homotypic adhesion and inhibits invasive characteristics of human breast cancer cells. Oncogene 24, 746–760 15592524 10.1038/sj.onc.1208203

[21] Peck A.R., Witkiewicz A.K., Liu C., Stringer G.A., Klimowicz A.C., Pequignot E. (2011) Loss of nuclear localized and tyrosine phosphorylated Stat5 in breast cancer predicts poor clinical outcome and increased risk of antiestrogen therapy failure. J. Clin. Oncol. 29, 2448–2458 21576635 10.1200/JCO.2010.30.3552 PMC3675698

[22] Nevalainen M.T., Xie J., Torhorst J., Bubendorf L., Haas P., Kononen J. (2004) Signal transducer and activator of transcription-5 activation and breast cancer prognosis. J. Clin. Oncol. 22, 2053–2060 15169792 10.1200/JCO.2004.11.046

[23] Koti M., Siu A., Clement I., Bidarimath M., Turashvili G., Edwards A. (2015) A distinct pre-existing inflammatory tumour microenvironment is associated with chemotherapy resistance in high-grade serous epithelial ovarian cancer. Br. J. Cancer 112, 1746 10.1038/bjc.2015.459 PMC4702007 26695556

[24] Rosen D.G., Mercado-Uribe I., Yang G., Bast R.C. Jr, Amin H.M., Lai R. (2006) The role of constitutively active signal transducer and activator of transcription 3 in ovarian tumorigenesis and prognosis. Cancer 107, 2730–2740 17063503 10.1002/cncr.22293

[25] Yang C., Lee H., Jove V., Deng J., Zhang W., Liu X. (2013) Prognostic significance of B-cells and pSTAT3 in patients with ovarian cancer. PLoS ONE 8, e54029 23326565 10.1371/journal.pone.0054029 PMC3542323

[26] Davidson B., Smith Y., Nesland J.M., Kaern J., Reich R. and Trope C.G. (2013) Defining a prognostic marker panel for patients with ovarian serous carcinoma effusion. Hum. Pathol. 44, 2449–2460 24011953 10.1016/j.humpath.2013.06.003

[27] Gyorffy B., Lanczky A., Eklund A.C., Denkert C., Budczies J., Li Q. (2010) An online survival analysis tool to rapidly assess the effect of 22,277 genes on breast cancer prognosis using microarray data of 1,809 patients. Breast Cancer Res. Treat. 123, 725–731 20020197 10.1007/s10549-009-0674-9

[28] Gyorffy B., Lanczky A. and Szallasi Z. (2012) Implementing an online tool for genome-wide validation of survival-associated biomarkers in ovarian-cancer using microarray data from 1287 patients. Endocr. Relat. Cancer 19, 197–208 22277193 10.1530/ERC-11-0329

[29] Gyorffy B., Surowiak P., Budczies J. and Lanczky A. (2013) Online survival analysis software to assess the prognostic value of biomarkers using transcriptomic data in non-small-cell lung cancer. PLoS ONE 8, e82241 24367507 10.1371/journal.pone.0082241 PMC3867325

[30] Blaszczyk K., Nowicka H., Kostyrko K., Antonczyk A., Wesoly J. and Bluyssen H.A. (2016) The unique role of STAT2 in constitutive and IFN-induced transcription and antiviral responses. Cytokine Growth Factor Rev. 29, 71–81 27053489 10.1016/j.cytogfr.2016.02.010

[31] Zhong Z., Wen Z. and Darnell J.E. Jr (1994) Stat3: a STAT family member activated by tyrosine phosphorylation in response to epidermal growth factor and interleukin-6. Science 264, 95–98 8140422 10.1126/science.8140422

[32] Sadowski H.B., Shuai K., Darnell J.E. Jr and Gilman M.Z. (1993) A common nuclear signal transduction pathway activated by growth factor and cytokine receptors. Science 261, 1739–44 8397445 10.1126/science.8397445

[33] Grabner B., Schramek D., Mueller K.M. and Moll H.P. (2015) Disruption of STAT3 signalling promotes KRAS-induced lung tumorigenesis. Nat. Commun. 6, 6285 25734337 10.1038/ncomms7285 PMC4366489

[34] Setsu N., Kohashi K., Endo M., Yamamoto H., Tamiya S., Takahashi Y. (2013) Phosphorylation of signal transducer and activator of transcription 3 in soft tissue leiomyosarcoma is associated with a better prognosis. Int. J. Cancer 132, 109–115 22644781 10.1002/ijc.27655

[35] Kaplan M.H. (2005) STAT4: a critical regulator of inflammation* in vivo*. Immunol. Res. 31, 231–242 15888914 10.1385/IR:31:3:231

[36] Hebenstreit D., Wirnsberger G., Horejs-Hoeck J. and Duschl A. (2006) Signaling mechanisms, interaction partners, and target genes of STAT6. Cytokine Growth Factor Rev. 17, 173–188 16540365 10.1016/j.cytogfr.2006.01.004

[37] Wang C.G., Ye Y.J., Yuan J., Liu F.F., Zhang H. and Wang S. (2010) EZH2 and STAT6 expression profiles are correlated with colorectal cancer stage and prognosis. World J. Gastroenterol. 16, 2421–2427 20480530 10.3748/wjg.v16.i19.2421 PMC2874149

